# Personalizing Therapy with Targeted Agents in Non-Small Cell Lung Cancer

**DOI:** 10.18632/oncotarget.245

**Published:** 2011-03-23

**Authors:** Rodrigo Dienstmann, Pablo Martinez, Enriqueta Felip

**Affiliations:** ^1^ Molecular Therapeutic Research Unit, Medical Oncology Service, Vall d'Hebron University Hospital and Vall d'Hebron Institute of Oncology, Barcelona; ^2^ Thoracic Neoplasms Unit, Medical Oncology Service, Vall d'Hebron University Hospital and Vall d'Hebron Institute of Oncology, Barcelona, Spain

**Keywords:** ALK, EGFR, KRAS, lung cancer, targeted therapy

## Abstract

In the last 6 years, since the first reports of an association between somatic mutations in epidermal growth factor receptor (*EGFR*) exons 19 and 21 and response to EGFR tyrosine kinase inhibitors (TKIs), treatment of non-small cell lung cancer (NSCLC) has changed dramatically. Based on laboratory and clinical observations, investigators have anticipated that these mutations could be predictive of response to EGFR TKIs and numerous studies have confirmed that the presence of mutation was associated with longer survival in patients receiving targeted therapy. Prospective trials comparing standard platinum-based chemotherapy with EGFR TKIs in patients with and without activating *EGFR* mutations validated the predictive value of molecular selection of patients for first-line treatment of advanced NSCLC. Recently, preclinical and first-in-human studies have demonstrated impressive activity of ALK TKI in tumors harboring *ALK* rearrangement. In this article, we review current data on molecular biology of lung cancer and evidence-based patient selection for targeted therapy.

## INTRODUCTION

Lung cancer is the most frequent cause of cancer-related death worldwide. Non-small cell lung cancer (NSCLC) accounts for 85% of all lung cancers [1]. Only a minority of NSCLC patients is suitable for radical treatment as curative care. Most patients have advanced disease at diagnosis and palliative therapy is the mainstay of management. Conventional chemotherapy of NSCLC has apparently reached a *plateau* of effectiveness in improving survival of lung cancer patients, and treatment outcomes must still be considered disappointing [2]. Based on a better understanding of the biology of lung cancer, targeted therapies are also available to treat patients with NSCLC. Predictive markers of response to these agents are undergoing prospective validation and promising results have been reported.

**Figure 1:**
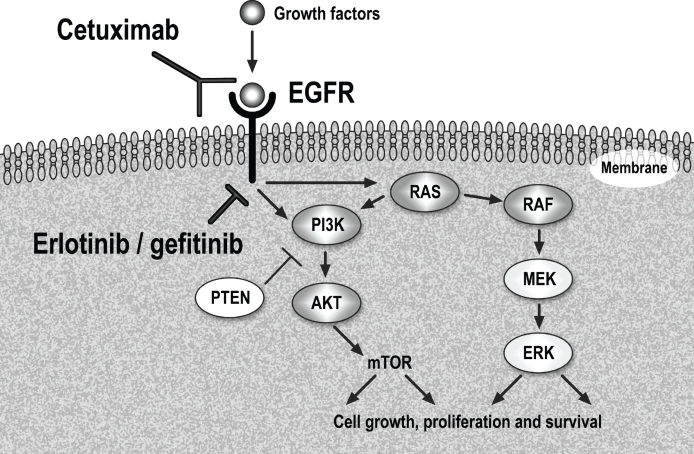
Epidermal Growth Factor Receptor (EGFR) pathway and anti-EGFR therapy in clinical use

The epidermal growth factor receptor (EGFR) signaling pathway is importantly implicated in tumor cell growth, local invasion, angiogenesis, metastasis, protein translation and cell metabolism. It activates two major pathways in solid tumors, RAS/RAF/MAPK/ERK and PI3K/AKT/mTOR, as seen in Figure 1 [3]. Molecular aberrations on the EGFR pathway are the most commonly studied predictive biomarkers of response/resistance to targeted agents in lung cancer. This review delineates the current role of EGFR inhibitors in the treatment of advanced NSCLC according to *EGFR* and *KRAS* status of the tumor, strategies to overcome resistance to agents targeting EGFR and also discusses other recently discovered molecular aberration in lung cancer, *ALK* rearrangement, which is being efficiently targeted with ALK inhibitors.

## EGFR PATHWAY: MOLECULAR ABERRATIONS, ANTI-EGFR THERAPY AND PREDICTIVE MARKERS OF RESPONSE

NSCLC is associated with EGFR overexpression in up to 80% of the patients and a high *EGFR* gene copy number is found in nearly 60% of the cases [4-6]. Mutation of the *EGFR* proto-oncogene is found in 10% to 20% of lung carcinomas (mostly adenocarcinomas) and nearly 90% of lung cancer-specific *EGFR* mutations comprise a leucine-to-arginine substitution at position 858 (L858R) and deletion mutations in exon 19 (delE746-A750) [7-10]. These mutations cause constitutive activation of the tyrosine kinase of the EGFR [11]. DNA sequencing is the most accurate method for identification of *EGFR* mutations in tissue samples. Using polymerase-chain reaction (PCR) amplification, deletions in exon 19 and exon 21 point mutations in codon 858 can be detected by length analysis and specific probes for wild-type and mutant sequences [12]. In addition, the Scorpion Amplified Refractory Mutation System (SARMS) technology can be used to detect *EGFR* mutations in serum genomic DNA or circulating lung-cancer cells [13, 14].

The EGFR kinase domain can be targeted with tyrosine kinase inhibitors (TKIs), such as erlotinib and gefitinib. In addition, another strategy to inhibit EGFR activity is with monoclonal antibodies such as cetuximab, a human-mouse chimeric IgG1 agent. It has been demonstrated that a subgroup of NSCLC patients achieves impressive response rates (RR), symptomatic improvement and long-term progression-free survival (PFS) with these agents. Since the first reports of an association between somatic mutations in *EGFR* exons 19 and 21 and response to gefitinib, treatment of NSCLC has changed dramatically [7, 8]. It has been shown that exon 19 deletions are more sensitive to erlotinib inhibition than the L858R mutation, a finding demonstrated by kinetic analysis [15] and also confirmed in clinical studies [16-18]. On the other hand, cetuximab is not as potent as EGFR TKIs in tumors with exon 19 deletion or L858R *EGFR* mutations [19].

Differences in the design of the clinical studies and technical approaches have led to some confusion about the role of molecular diagnostics in guiding the use of EGFR-targeted therapy in NSCLC. Most information regarding clinical benefit with these agents comes from retrospective analysis of large studies. At the present time, prospective clinical data confirming the predictive value of receptor mutations for response to EGFR TKIs is available [20, 21]. Apart from mutation analysis, EGFR protein expression determined by immunohistochemistry (IHC) and *EGFR* gene copy number determined by fluorescent *in situ* hybridization (FISH) have been evaluated as markers for clinical decision making regarding EGFR TKI therapy. Technical considerations are important in assessing IHC, which suffers from the lack of a standard methodology and inconsistencies among testing centers [6]. In addition, gene copy number evaluation by FISH may be affected by tumor heterogeneity within analyzed specimens. Therefore, a detailed review of the clinical trials evaluating molecular markers of response to anti-EGFR agents is warranted.

## REVIEW OF CLINICAL TRIALS WITH ANTI-EGFR AGENTS IN NSCLC

### Gefitinib and Erlotinib

Phase I studies of gefitinib defined dose-limiting toxicities at 700 to 1000 mg/day [22, 23]. However, pharmacodynamic data showed that a dose of 150 mg/day was sufficient to suppress EGFR signaling in skin biopsy specimens [24]. As chronic daily doses higher than 500 mg/day were relatively not well tolerated, further studies evaluated 250 and 500 mg/day doses. Gefitinib was examined as monotherapy in two phase II studies called IDEAL trials [25, 26]. Response rates with doses of 250 and 500 mg/day were similar, ranging from 10% to 18%. Notably, responses were more likely in patients with specific characteristics: Asians, females, non-smoking and those with adenocarcinoma tumors. Posterior analysis demonstrated that patients with and *EGFR* mutation had an improved RR to gefitinib compared to wild-type patients (46% versus 10%), as shown in Table 1 [27].

**Table 1:** Response rate to EGFR tyrosine kinase inhibitors according to EGFR mutation status

	EGFR mutation (+)	EGFR mutation (−)	Reference
	% (patients)	% (patients)	
**Gefitinib**			
*Retrospective evaluation*			
IDEAL trial	46% (13)	10% (56)	27
ISEL trial	37% (16)	2% (116)	30
INTEREST trial	42% (19)	NR	32
*Prospective evaluation*			
Inoue et al.	75% (16)	-	43
Sequist et al.	55% (31)	-	45
Kim et al.	53% (45)	-	18
**Erlotinib**			
*Retrospective evaluation*			
BR.21	27% (15)	7% (101)	36
Janne et al.	66% (32)	8% (48)	47
*Prospective evaluation*			
Spanish Lung Cancer trial	70% (217)	-	16
NR: not reported			

The early trials that evaluated EGFR TKIs for the second- and third-line settings of advanced NSCLC did not select patients on the basis of any EGFR marker [28, 29]. The ISEL trial evaluated the role of second-line gefitinib 250 mg/day in 1,692 patients with advanced NSCLC [29]. Median overall survival (OS) was 5.6 months, compared to 5.1 months in the placebo group, difference not statistically significant. High EGFR gene copy number (30.8% of patients) was associated with a nonsignificant trend toward improved OS with gefitinib treatment and patients with EGFR mutations had higher RR than patients without (37.5% versus 2.6%) [30]. In the INTEREST trial, 1,466 pretreated patients with advanced NSCLC were randomly assigned to receive gefitinib or docetaxel in the second-line setting [31]. Non-inferiority of gefitinib compared to docetaxel was confirmed for OS (7.6 versus 8.0 months). EGFR mutation-positive patients had significantly longer PFS (HR “ Hazard Ratio = 0.16) and higher objective RR (42.1% versus 21.1%) with gefitinib compared to docetaxel. Patients with high EGFR copy number also had higher RR with gefitinib (13% versus 7.4%) [32].

In contrast to gefitinib, dose of erlotinib studied in phase II and III trials is the maximum-tolerated dose defined in phase I trials (150 mg/day) [33]. A phase II trial in patients with refractory NSCLC confirmed RR of approximately 12% and survival outcomes similar to gefitinib [34]. The BR.21 study (erlotinib in second- and third-line settings of advanced NSCLC) found superiority in survival with erlotinib group as compared to placebo (median OS of 6.7 versus 4.7 months) [28]. Molecular analysis of BR.21 trial showed that FISH positivity (45% of tumors) was predictive of improved survival with erlotinib (HR = 0.44) [35]. *EGFR* mutations were confirmed in 17% of tumors (34 patients had classical *EGFR* activating mutations). Response rate was significantly higher in patients with these mutations (27% versus 7%) compared to patients with wild-type or other indeterminate mutations [36]. This trial also confirmed symptomatic and survival benefit of erlotinib in patients without initial clinical predictors of response to EGFR TKIs [37]. One possible explanation to this finding is that erlotinib was administered at its maximum-tolerated dose, which could have different effects in the population of patients with wild-type *EGFR*, not hypersensitive to EGFR TKIs. This correlates with the higher prevalence of rash, a known marker of efficacy with these agents, in the erlotinib trial (76%, as compared to 37% in the gefitinib trial) [38].

Another study with relevant molecular data is the SATURN trial, which randomly assigned 889 patients with advanced NSCLC who had response or stable disease after four cycles of platinum-based chemotherapy to erlotinib or placebo as maintenance treatment [39]. Progression-free survival was significantly improved in the erlotinib maintenance arm (HR = 0.71). Tumor biomarker analysis of *EGFR* mutation status was available for 437 patients: those with *EGFR* mutation had median PFS of 44.6 weeks on erlotinib compared to 13 weeks with placebo (HR = 0.1), a remarkable benefit for this subgroup of patients. *EGFR* mutation was the only marker significantly predictive of differential erlotinib effect [39].

### Cetuximab

In the FLEX trial, 1,125 patients with advanced NSCLC and EGFR-positive tumors by IHC were randomly assigned to chemotherapy plus cetuximab or chemotherapy alone [40]. Patients given chemotherapy plus cetuximab survived longer than those in the chemotherapy-alone group (median OS of 11.3 versus 10.1 months; HR for death 0.87, p = 0.044). Molecular biomarker study showed that no significant differences in survival were detected between patients with FISH positive or negative tumors in either treatment arm [41]. As anticipated by preclinical data, *EGFR* mutation status (positive in 17% of cases) was not predictive of benefit with cetuximab. However, the prognostic value of mutation was confirmed, with longer overall survival in patients with *EGFR* mutation tumors compared to wild-type tumors in both treatment arms [41].

A similar trial was carried out in the USA in 676 nonselected patients with advanced NSCLC (BMS099) [42]. There was no survival benefit with the addition of cetuximab to paclitaxel-carboplatin combination chemotherapy. Tissue samples for biomarker subanalysis were available for one-third of the patients. EGFR expression, *EGFR* copy number and *EGFR* mutations were not associated with treatment outcomes [42].

## PROSPECTIVE STUDIES WITH EGFR-TKIS IN SELECTED PATIENT POPULATIONS

Studies with first-line EGFR TKIs in advanced lung cancer have been recently reported. The first study was conducted in Asian patients and examined EGFR status in 75 patients [43]. Sixteen patients with EGFR mutation were enrolled onto the study and received gefitinib 250 mg/day. Overall RR was 75% and median PFS was 9.7 months. As seen in Table 1, a confirmatory trial with gefitinib as first-line treatment of advanced EGFR mutant NSCLC reported RR of 53% in 45 patients [18]. Interesting data came from another prospective trial in Japan that enrolled 30 patients with EGFR mutations and poor Performance Status (PS) without indication for palliative chemotherapy [44]. The overall RR was 66% and the disease control rate was 90%. Of note, 15 of 22 patients with baseline PS 3 improved to PS 1. Median PFS and OS were 6.5 months and 17.8 months, respectively [44].

Prospective screening studies in non-Asian populations have also been reported. The first study screened chemotherapy-naïve patients with advanced NSCLC and clinical characteristic associated with EGFR mutations [45]. Response rate was 55% in 31 patients who received gefitinib and the median PFS was 9.2 months. The authors also evaluated EGFR gene copy number by FISH and concluded that it did not provide additional predictive information. This was because most patients who harbor EGFR mutations also had high gene copy numbers [46]. The Spanish Lung Cancer Group trial observed a prevalence of EGFR mutations in patients with lung cancer in Spain around 16% (350 of 2,105 cases) [16]. In 60% of the patients, EGFR mutations were also detected in the serum. Median PFS and OS for 217 patients who received erlotinib were 14 months and 27 months, respectively, and radiologic RR was around 70%. Duration of response was similar for patients receiving first-line therapy (14 months) or second-line therapy (13 months) [16].

Recently, results of the phase II randomized trial of erlotinib compared to erlotinib in combination with paclitaxel-carboplatin chemotherapy in treatment-naïve, never or light former smokers with advanced lung adenocarcinoma [47]. Of the 182 patients randomized, 67 (39%) had mutant and 105 (61%) wild-type EGFR. In the overall patient population, addition of chemotherapy did not significantly increase response rate or PFS. EGFR mutant patients had median PFS of 15.7 months with single-agent erlotinib as compared to 17.2 months with combination therapy. On the other hand, PFS in patients with tumors harboring wild-type EGFR was only 2.4 months with single-agent erlotinib and 4.8 months with the addition of chemotherapy [47].

Based on these findings, several phase III trials are comparing first-line treatment of advanced NSCLC with chemotherapy or EGFR TKI in specific populations of patients: (a) in a clinically-enriched subgroup; or (b) in a biomarker-selected subgroup - those with documented EGFR exon 19 or 21 mutations. The first published trial, named IPASS, selected more than 1,200 patients to receive gefitinib 250 mg/day or standard paclitaxel-carboplatin chemotherapy [17]. Only patients with clinical characteristics predictive of response to EGFR TKIs were enrolled (adenocarcinoma histology; nonsmokers, defined as patients who had smoked <100 cigarettes in their lifetime; or former light smokers, defined as those who had stopped smoking at least 15 years previously and had a total of ≤10 pack-years of smoking). The study met its primary objective of showing the noninferiority of gefitinib and also showed its superiority, as compared to chemotherapy, with regards to PFS (HR for progression or death = 0.74). The 12-months rates of PFS were 24.9% with gefitinib and 6.7% with paclitaxel-carboplatin. However, the overall population result is clearly of less relevance than the outcome in subgroups of patients. As shown in Table 2, in the 261 patients who were positive for the EGFR mutation, PFS was significantly longer among those who received gefitinib than among those who received paclitaxel-carboplatin (HR = 0.48), whereas in the subgroup of 176 patients who were negative for the mutation, PFS was significantly longer among those who received chemotherapy (HR for progression or death with gefitinib = 2.85). In the mutation-negative population, objective RR was only 1.1% with gefitinib versus 23.5% with chemotherapy. High EGFR copy number by FISH was predictive for efficacy only when accompanied by the presence of concomitant EGFR mutation. Patients with a high EGFR copy number and wild-type EGFR did not benefit from gefitinib, but did benefit from chemotherapy [17]. No difference in OS was seen in patients with EGFR mutation regardless of the treatment assigned [48]. The major reason for the lack of survival benefit is the cross-over effect, as half of the patients in the chemotherapy arm received EGFR TKIs at disease progression [48]. The second trial assigned 309 chemotherapy-naïve patients with adenocarcinomas who had never smoked to receive 250 mg gefitinib daily or gemcitabine plus cisplatin at standard doses [49]. Progression-free survival was similar in both arms (6.1 months for gefitinib, 6.6 months for chemotherapy) and in the subgroup of patients with known EGFR mutations (44%), gefitinib produced a higher RR, as shown in Table 2 [49]. Both studies emphasize the importance of molecular selection of patients for first-line treatment with an EGFR TKI.

**Table 2:** Prospective randomized studies of first-line EGFR TKIs versus standard chemotherapy in clinically-enriched and biomarker-selected patient populations

Study	Population	Treatment Arms	Response Rate	Progression-free survival (favoring EGFR TKI)	Overall survival	Reference
IPASS	Asians, never/former light smokers, adenocarcinoma (*216 patients with EGFR mutation)*	Gefitinib × Paclitaxel/Carboplatin	71% × 47%	HR 0.48 (0.36 - 0.64)	HR 1.0 (0.76-1.33)	17
First-SIGNAL	Asians, never smokers, adenocarcinoma *(26 patients with EGFR mutation received gefitinib)*	Gefitinib × Gemcitabine/Cisplatin	85% × 37%	8.4 × 6.7 months (NS)	Not available	49
North-East Japan	*EGFR* mutation, 200 patients	Gefitinib × Paclitaxel/Carboplatin	74% × 31%	10.8 × 5.4 months HR 0.31 (0.22 - 0.41)	30.5 × 23.6 months (NS)	20
WJTOG3405	*EGFR* mutation, 177 patients	Gefitinib × Docetaxel/Cisplatin	62% × 32%	9.2 × 6.3 months HR 0.49 (0.34-0.71)	Not available	21
OPTIMAL	*EGFR* mutation, 165 patients	Erlotinib × Gemcitabine/Carboplatin	83% × 36%	13.1 × 4.6 months HR 0.16 (0.10-0.26)	Not available	50
EURTAC	*EGFR* mutation, approximately 170 patients	Erlotinib × Platinum doublet	Pending	Pending	Pending	
LUX-Lung 3/LUX-Lung 6	*EGFR* mutation, approximately 330 patients each study	BIBW2992 × Pemetrexed/Cisplatin or Gemcitabine/Cisplatin	Pending	Pending	Pending	

Preliminary results of other phase III trials that randomized patients with metastatic NSCLC and *EGFR* mutations at baseline to receive EGFR TKIs or standard first-line platinum-based regimens are presented in Table 2. The North-East Japan trial (prematurely interrupted after interim analysis) and the WJTOG3405 trial demonstrated that treatment with gefitinib doubles the RR and significantly improves PFS as compared to chemotherapy [20, 21]. The first study with erlotinib as comparator was recently presented. In the OPTIMAL trial, Asian patients with chemonaïve NSCLC with *EGFR* mutations were randomized to erlotinib 150 mg/day or gemcitabine plus carboplatin [50]. Both RR and PFS were higher in the EGFR TKI subgroup [50]. Additional prospective studies are underway, as seen in Table 2.

## OVERCOMING RESISTANCE TO EGFR TKIS

Tumors become resistant when they reactivate downstream signaling despite the presence of the EGFR inhibitor. Zhang et al. summarize the current understanding of the functional role of activating *EGFR* mutations in addition to the pivotal primary and acquired resistance mechanisms to EGFR inhibitors [51]. Resistance is typically caused by mutations in the *EGFR* gene that are not associated with sensitivity to EGFR TKIs, such as insertion mutations in exon 20 [52], or by other somatic mutations in genes that have an impact on the EGFR signaling pathway, such as *KRAS* [53]. Acquired resistance may be caused by additional mutations in the *EGFR* gene obtained during the course of treatment that change the protein-coding sequence or by amplification of another oncogene signaling pathway [54].

The most commonly identified mechanism of resistance is an *EGFR* mutation at position 790 (T790M), resulting in substitution of a threonine residue with methionine, which abrogates the ability of gefitinib or erlotinib to inhibit EGFR [55, 56]. This mutation can be found in 50% of the tissue samples from patients with acquired gefitinib resistance [57]. Another acquired mutation in *EGFR*, which leads to substitution of alanine for threonine at position 854 (T854A) and hinders the inhibition of tyrosine phosphorylation by erlotinib, has also been reported [58]. Various irreversible EGFR inhibitors, such as BIBW2992/afatinib (targeting EGFR and HER2) and PF00299804 (targeting EGFR, HER2 and HER4) are undergoing clinical development. These agents may prevent and overcome primary and acquired resistance to first-generation reversible EGFR TKIs. In the LUX-Lung 2 study, 129 patients with activating *EGFR* mutations and no previous EGFR TKI therapy received BIBW2992 as single agent [59]. Overall RR was 60%, with a promising PFS of 14 months. This drug has also shown activity in patients whose tumors harbored less common *EGFR* mutations [60]. Its efficacy was also evaluated as a rescue treatment after failure to erlotinib or gefitinib in a randomized phase III trial [61]. At primary analysis (358 events in 585 patients), median OS was 10.8 months in the BIBW2992 group and 11.9 months in the placebo group. A significant PFS increase was seen in patients that received the study drug (3.3 versus 1.1 months) [61]. Regarding the PF00029804 compound, preliminary data from a phase II randomized trial showed that PFS was superior when compared to erlotinib in the general population of patients with chemotherapy-refractory NSCLC, not selected according to *EGFR* mutation status (12.4 weeks versus 8.3 weeks) [62]. As first-line treatment of patients with known *EGFR* mutation or clinically selected (Asians with adenocarcinoma and non-or light smoking history), PF00029804 showed encouraging efficacy, with 6 month-PFS rate of 67% (85% in those with *EGFR* mutation) [63].

Activation of downstream signaling via alternative mechanisms that stimulate the RAS/RAF/MAPK/ERK and PI3K/AKT/mTOR pathways is another mechanism of resistance to EGFR TKIs. This occurs with activation of the IGF-1R pathway (Insulin-like Growth Factor 1 Receptor) [64], amplification/mutations of MET (the receptor for hepatocyte growth factor receptor, identified in 10-20% of NSCLC) [65-67], and *PIK3CA* amplification/mutations (identified in up to 17% of NSCLC) [68]. In this situation, there appears to be dual input to signaling and combined inhibition of EGFR and the alternative pathway may be necessary to kill tumor cells. A combination approach may prevent the emergence of resistance that eventually occurs following initial response to EGFR TKIs and may increase the proportion of *EGFR* wild-type patients that respond. Preliminary data of a phase II randomized trial of erlotinib and placebo or in combination with a non-ATP competitive receptor TKI of c-MET (ARQ 197) were recently presented [69]. Progression-free survival was significantly higher for the dual inhibition approach (HR = 0.68, p < 0.05) [69]. A monoclonal antibody targeting MET (MetMab) is also under clinical development. In patients with advanced NSCLC and MET expression by IHC analysis, the combination of erlotinib and MetMab significantly increased PFS (HR = 0.56, p = 0.05) as compared to erlotinib and placebo in a phase II randomized trial [70]. Multiple phase I and II trials are underway to evaluate the additive benefit of other targeted agents, such as anti-IGF1R monoclonal antibodies, PI3K/mTOR and MEK inhibitiors.

## *KRAS* STATUS AND RESPONSE TO ANTI-EGFR AGENTS

*KRAS* mutations were identified in NSCLC tumors more than 20 years ago [71]. *KRAS* encodes a GTPase downstream of EGFR and mutations (most frequent in exons 12, 13 and 61) lead to stimulus-independent, persistent activation of downstream effectors of the RAF/MAPK/MEK/ERK cascade [72]. They are associated with significant tobacco exposure and worse prognosis, although contradictory data have been reported [73, 74]. Prevalence of mutation is about 20% in the overall lung cancer patient population, 5% to 15% in never-smokers patients with adenocarcinoma and approximately 5% in the squamous cell carcinoma subtype [75-77].

KRAS mutations are associated with primary resistance to EGFR TKIs [78]. Phase II trials have shown very small or absent response rates to erlotinib in patients with KRAS mutations [79]. A recent meta-analysis demonstrated 3% rate of objective tumor response to EGFR TKIs in patients with KRAS mutations, as compared to 26% in those with wild-type KRAS [80]. Data from the TRIBUTE trial (chemotherapy with or without erlotinib for previously untreated patients with NSCLC) suggest that, in KRAS mutated patients, OS and RR may be worse with the addition of EGFR TKI [81]. KRAS mutant patients (20% of available tumor samples) showed a RR of 8% with paclitaxel-carboplatin plus erlotinib, compared with 23% for patients that received the chemotherapy doublet alone. Of note, the RR for patients treated with chemotherapy alone did not differ significantly by KRAS mutation status (26% in those without mutation versus 23%) [81]. In the INTEREST trial, *KRAS* mutation was not a predictive factor for a differential survival effect between gefitinib and docetaxel [32]. In the BR.21 trial, significant survival benefit from second- or third-line erlotinib therapy was observed for patients with wild-type KRAS (HR = 0.69, p=0.03) but not for patients with mutant KRAS (HR = 1.67, p = 0.31) [36]. Molecular data from the maintenance SATURN trial suggested that KRAS mutation was a negative prognostic factor for patients receiving placebo, with significantly shorter PFS (HR = 1.5, p = 0.017) [39]. Patients with wild-type KRAS had statistically significant benefit with erlotinib in terms of PFS (HR = 0.7, p = 0.0009). The hazard ratio for PFS was similar in the KRAS mutant population, but the benefit was not statistically significant (HR = 0.77, p = 0.22) [39].

Based on data from *KRAS* mutation status and benefit of cetuximab in advanced colon cancer, molecular analysis of FLEX trial was conducted. Of the 1,125 patients enrolled, 395 had tumor samples available and *KRAS* mutation was detected in 19%. The comparison of the cetuximab treatment effects in patients with *KRAS* wild-type tumors and those with *KRAS* mutant tumors showed no marked differences with regard to PFS or OS [41]. In this trial, the benefit of cetuximab was observed regardless of *KRAS* mutational status. The same results were obtained with retrospective analysis of the BMS099 trial [42]. Therefore, *KRAS* status does not predict sensitivity to cetuximab in NSCLC.

## ALK REARRANGEMENT AND TARGETED THERAPY

A fusion gene between echinoderm microtubule-associated protein like 4 (*EML4*) and the anaplastic lymphoma kinase (*ALK*) has recently been identified in NSCLC [82]. Although more frequent in hematological malignancies, recurrent chromosome translocations may play a role in the molecular pathogenesis of solid tumors. The fusion gene *EML4-ALK*, inv(2)(p21p23), becomes activated to exert a marked oncogenicity both *in vitro* and *in vivo,* possibly by switching on the RAS/RAF signaling pathway [83]. *ALK* rearrangement can be identified by FISH analysis using break-apart probes to ALK, which detects disruption of the *ALK* locus but does not confirm *EML4* as the partner fusion gene. Recently, a novel highly sensitive antibody allowed for the routine detection of ALK-rearranged lung carcinomas by standard IHC [84].

Among 266 resected NSCLCs in an East Asian population, the EML4-ALK fusion gene was found in about 5% of cases, as assessed by reverse transcriptase-PCR and posterior sequencing [85]. EML4-ALK was associated with younger age of cancer onset and with never-smoking status. EML4-ALK, EGFR, and KRAS mutations were all mutually exclusive, suggesting that ALK mutation may be an important oncogenic factor, and a potential therapeutic target in EGFR wild-type and KRAS wild-type lung cancer. When patients are selected for genetic screening on the basis of two or more of clinical characteristics (female sex, Asian ethnicity, never/light smoking history, and adenocarcinoma histology), a recent study demonstrated that among 141 tumors evaluated, 19 (13%) were EML4-ALK mutant and 31 (22%) were EGFR mutant [86]. Considering only never/light smokers without EGFR mutation, the frequency of EML4-ALK was 33%. EML4-ALK positivity was related with resistance to EGFR TKIs but similar RR to platinum-based combination chemotherapy. In addition, presence of EML4-ALK mutation was not associated differences in OS, as compared to those patients with wild type EML4-ALK and EGFR [86].

Impressive clinical activity was demonstrated in a phase I trial of an oral ATP- competitive TKI of ALK and c-MET, crizotinib (PF-02341066), in patients with advanced NSCLC and whose tumors harbored *ALK* rearrangement by FISH analysis [87]. Patients enrolled shared several key clinical features with *EGFR*-mutated patients (adenocarcinoma histology and nonsmoking history). Among 82 patients treated in an expanded cohort of the dose-escalation study (250 mg twice daily), 57% had an objective RR and 72% were progression-free at 6 months [87]. All patients tested negative for *EGFR* mutation and amplification of MET, another target for crizotinib, which suggested that the therapeutic effect is through inhibition of ALK. Further confirmatory studies are underway, along with a phase III trial comparing treatment of *ALK* rearrangement positive patients with crizotinib or standard chemotherapy in the second-line setting.

## CONCLUSIONS

Identifying the patients who are most likely to obtain clinical benefit from targeted therapies in NSCLC is of paramount importance. To make rational clinical decisions, in addition to understanding the biology of the disease, oncologists need to rely on standardized and validated methods of molecular assessment. *EGFR* amplification by FISH and protein expression measured by IHC are not informative for personalized therapy in advanced NSCLC but further analysis of studies that combine chemotherapy with monoclonal antibodies targeting EGFR is indicated. In addition, the value of determining *KRAS* mutation status to select EGFR TKI therapy is not clear. On the other hand, it is now well established that specific genetic lesions that drive the proliferation of cancer cells render some tumors very sensitive to therapeutic inhibitors targeting the mutated pathway. The most useful biomarker is *EGFR* mutation status and its determination is mandatory for proper therapeutic decisions. It is a good prognostic factor and has also predictive value for selecting treatment with EGFR TKIs. Different from the initially unsuccessful trials of EGFR inhibitors in nongenotyped patients, data from multiple phase III trials show superiority of gefitinib/erlotinib over standard chemotherapy for advanced NSCLC in terms of RR and PFS in the biomarker-selected *EGFR* mutation positive subgroup of patients. Additionally, *ALK* rearrangement is a promising biomarker of benefit with ALK inhibitors and its detection resulted in prompt translation of preclinical data to patient care. Only 3 years after the initiation of the phase I trial, a phase III registration study of crizotinib in *ALK*-positive patients started enrollment. Participation in a clinical trial is the best alternative for this subgroup of patients. Importantly, although *EGFR* and *ALK* mutations are found mostly in patients with history of no smoking or light smoking who have adenocarcinoma, genotyping should be offered to all patients with advanced NSCLC if treatment with specific TKIs is available.

In conclusion, EGFR TKI therapy should be recommended to patients with activating *EGFR* mutations in the course of the disease. However, the lack of OS advantage with early treatment raises the question of whether we should give these drugs up-front, as maintenance therapy or as second-/third-line options. Clinical practice in the treatment of advanced NSCLC patients has shown that progression and symptomatic deterioration can occur very short after treatment discontinuation. EGFR TKI therapy may be started once the mutation status is known: (a) as first-line therapy in all patients, especially those unfit or who do not agree with chemotherapy; and (b) as maintenance/ “early second-line” therapy for patients that received previous chemotherapy. Erlotinib may be considered a salvage treatment in unselected patient populations of chemotherapy-refractory NSCLC, as disease stabilization and symptomatic improvement was observed independent of molecular or clinical predictors of benefit. In addition, more clinical and translational data on irreversible EGFR inhibitors and dual targeted therapy in molecularly-selected subgroups of patients will help oncologists to personalize therapy of advanced NSCLC even further.
